# 2-Azidobenzaldehyde-Based [4+2] Annulation for the Synthesis of Quinoline Derivatives

**DOI:** 10.3390/molecules29061241

**Published:** 2024-03-11

**Authors:** Xiaofeng Zhang, Miao Liu, Weiqi Qiu, Wei Zhang

**Affiliations:** 1 Center for Green Chemistry and Department of Chemistry, University of Massachusetts Boston, 100 Morrissey Blvd, Boston, MA 02125, USA; liumiaomarcus@gmail.com (M.L.); qiuwb@bc.edu (W.Q.); 2 Department of Medicinal Chemistry, Cerevel Therapeutics, Cambridge, MA 02141, USA; 3 Department of Mechanical Engineering, University of Wisconsin Milwaukee, Milwaukee, WI 53211, USA; 4 Department of Chemistry, Boston College, 2609 Beacon Street, Chestnut Hill, MA 20467, USA

**Keywords:** quinolines, azidobenzaldehyde, [4+2] annulation, heterocyclic, bioactive

## Abstract

Quinoline is a privileged heterocyclic ring which can be found in many drug molecules and bioactive compounds. The development of synthetic methods for making quinoline derivatives continuously attracts the interest of organic and medicinal chemists. This paper highlights 2-azidobenzaldehyde-based [4+2] annulation for the synthesis of quinoline derivatives including fused and spiro-quinolines, quinoline-4-ols, 4-aminoquinolines, and related compounds.

## 1. Introduction

**Figure 1:**
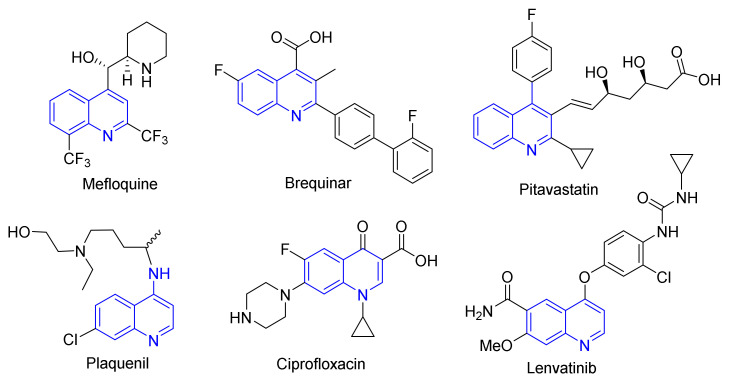
Representative structures of quinoline drugs.

Quinoline is a privileged scaffold for bioactive molecules [1,2,3,4,5,6]. Shown in Figure 1 are some quinoline-containing drug molecules, including Mefloquine (antimalarial) [7,8,9,10], Brequinar (anticancer) [11,12,13], Pitavastatin (cholesterol-lowering) [14,15,16], Plaquenil (antimalarial) [17,18], Ciprofloxacin (antibacterial) [19,20,21], and Lenvatinib (anticancer) [22,23,24]. Other than these commercial drugs, a great number of quinoline derivatives have been reported or are under development as druggable molecules for anticancer [25,26], antibacterial [27,28,29], antifungal [30,31,32], antiviral [33,34,35,36], antimalarial [37,38,39], antioxidant [40,41,42], anti-inflammatory [43,44,45], CNS effect [46,47], cardiovascular, anticonvulsant, analgesic, and anthelmintic research [48,49,50,51].

The synthesis of quinolines has continuously attracted the interest of organic and medicinal chemists. Over the years, many methods including name reactions such as Skraup, Doebner–von Miller, Friedlander, Ptzinger, Conrad–Limpach, and Combes syntheses [52,53] have been developed for making quinolines. Alam and Patel have reviewed the general synthetic methods [54,55], which included new methods such as cascade reactions [56] and metal-free one-pot synthesis [57,58].

**Scheme 1:**
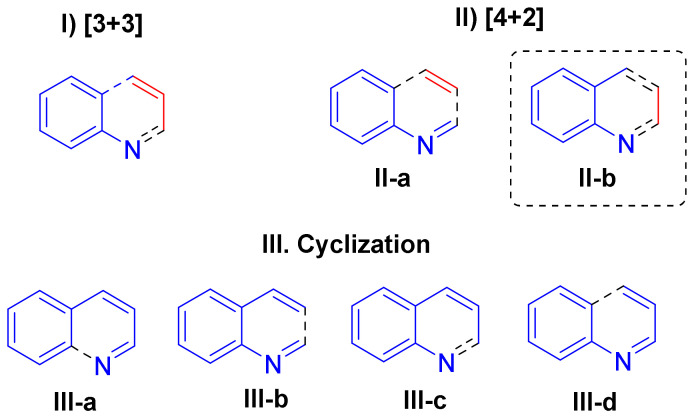
Common synthetic strategies for making quinolines.

Shown in Scheme 1 are three general approaches to assembling the quinoline ring. (**I**) [3+3] Annulation. The early developed methods such as the Skraup, Doebner–von Miller, Conrad–Limpach, Gould–Jacobs, and Combes syntheses are in this category. The drawback of this approach is that it has a low regioselectivity for the synthesis of multi-substituted quinolines [52,54,57]. (**II**) [4+2] Annulation. There are two ways to put together the pyridine ring according to [4+2] annulation (**II-a** and **II-b**). The first one has low regioselectivity and a limited substrate scope [52,55,56]. The second one is more popular and uses readily available substrates to give products with a good regioselectivity. Reactions such as the Friedlander and Ptzinger reactions are in this category [52,53,54,55,58]. (**III**) Cyclization. In theory, there are four different ways to form the pyridine ring of quinolines (**III-a** to **III-d**) according to cyclization. But they have not been fully developed due to the difficult reaction process and the limited availability of the substrates [59,60].

Presented in this paper are azidobenzaldehyde-based [4+2] annulation reactions (**II-b** type) for the synthesis of substituted quinolines [61]. For example, [4+2] annulation could be accomplished using a direct Diels–Alder reaction or via sequential condensation and cyclization reactions. In addition to quinolines, versatile 2-azidobenzaldehydes **1** could be used for the synthesis of other heterocyclic rings such as indoles [62,63], 3,4-dihydroquinazolines [64,65], triazolobenzodiazepines [66], *2H*-indazoles [67], benzoxazepinones [68], and quinolines. Meanwhile, 2-azidobenzaldehydes could be easily prepared from commercially available 2-halobenzaldehydes, 2-aminobenzaldehydes, and 2-nitrobenzaldehydes [62,64,66,67,68]. Over 30 papers on the 2-azidobenzaldehyde-based synthesis of quinolines could be found in the literature and are summarized in this paper.

## 2. Results

### 2.1. Synthesis of 4-Unsubstituted Quinolines

**Figure 2:**
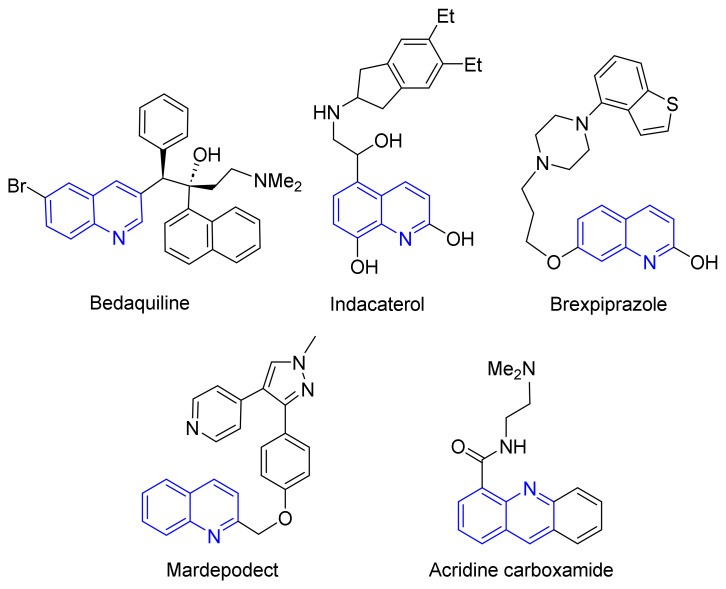
Examples of 4-unsubstituted quinoline-based marketed drugs and clinical candidates.

There are many 4-nonsubstituted quinoline drugs and drug candidates (Figure 2), such as Bedaquiline for the treatment of active tuberculosis [69,70], Indacaterol for the treatment of chronic obstructive pulmonary disease [71,72], Brexpiprazole for the treatment of schizophrenia [73], Mardepodect for the treatment of schizophrenia [74], and Acridine carboxamide for the treatment of cancer [75].

**Scheme 2:**
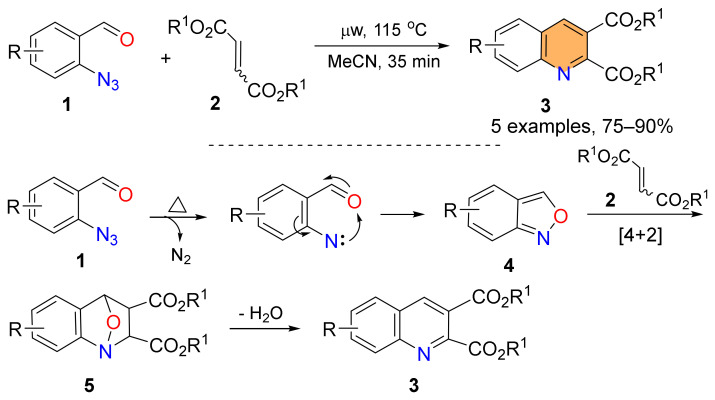
Cascade reactions for quinolinedicarboxylates **3**.

Utilizing the Diels–Alder reaction of benzisoxazole [76,77,78] as a key step, Zhang and co-workers developed a one-pot reaction involving the denitrogenation of 2-azidobenzaldehyde **1**, the formation of benzisoxazole **4**, [4+2] cycloaddition with fumarate ester **2**, and, finally, dehydrative aromatization of intermediate **5** for the synthesis of quinolinedicarboxylates **3** (Scheme 2) [79].

**Scheme 3:**
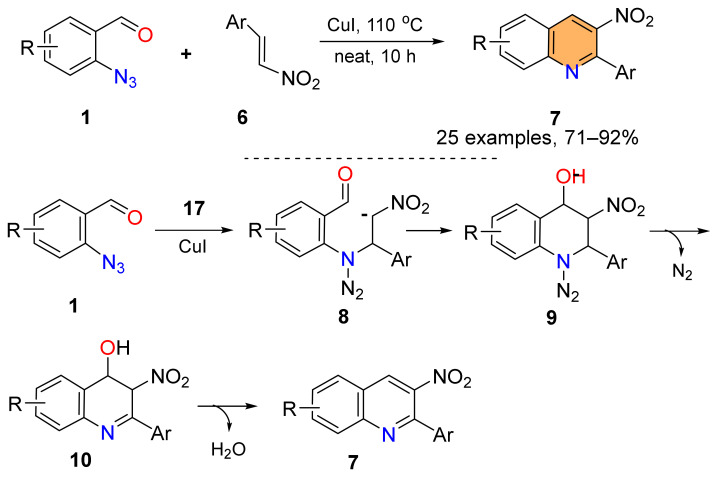
Cu-catalyzed cascade reaction for 3-NO_2_ quinolines **7**.

Zeng and Chen reported the Cu-catalyzed synthesis of 3-NO_2_ quinolines **7**. The addition of nitroalkene **6** to the azide group of **1** affords **8**, which then undergoes cyclization to form **9**. Proton transfer and the release of N_2_ gas from **9** affords **10**, which leads to the formation of 3-NO_2_ quinolines **7** after dehydration (Scheme 3) [80].

**Scheme 4:**
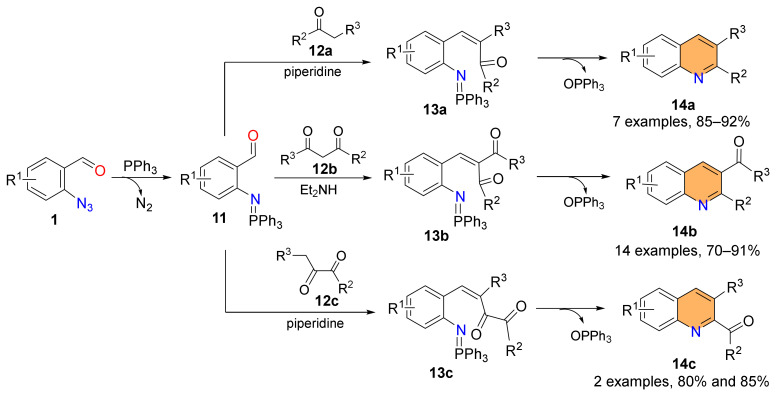
Cascade synthesis of 2,3-substituted quinolines **14a**–**c**.

The intramolecular aza-Wittig reaction can be used for making *N*-heterocyclics [81,82]. Zhang and He reported the synthesis of 2,3-substituted quinolines **14** through a sequence involving the Staudinger, Knoevenagel, and aza-Wittig reactions [83,84]. The first intermediates **11** generated according to the Staudinger reaction of 2-azidobenzaldehydes **1** and PPh_3_ undergo the Knoevenagel reaction with different carbonyl compounds **12a**–**c** to generate the corresponding intermediates **13a**–**c**. The intramolecular aza-Wittig reaction releasing Ph_3_P=O gives 2,3-substituted quinoline products **14a**–**c** (Scheme 4).

**Scheme 5:**
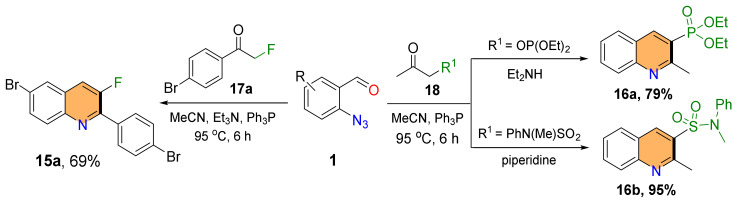
Synthesis of 3-F quinolines **15a**, 3-phosphonylquinolines **16a**, and 3-sulfonylquinoline **16b**.

**Scheme 6:**
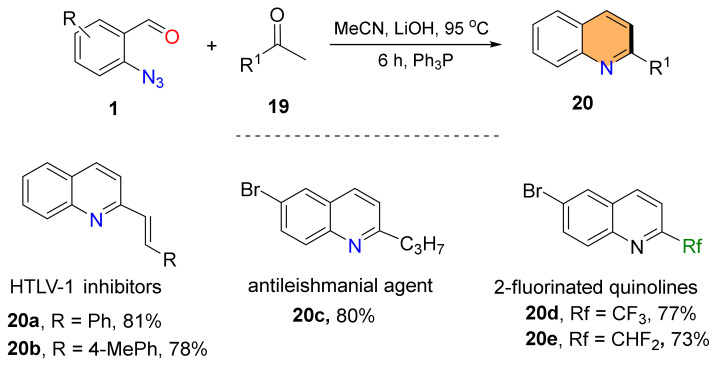
Synthesis of bioactive 2-substituted quinolines **20**.

Zhang and co-workers expanded the scope of the aza-Wittig reaction for synthesizing 3-F quinoline **15a**, 3-phosphonylquinoline **16a**, and 3-sulfonylquinoline **16b** using **1** and **17a** or **18** as the substrates (Scheme 5) [83,85]. They also used 2-azidobenzaldehydes **1** and 1-substituted propan-2-ones **19** in the synthesis of bioactive 2-substituted quinolines such as HTLV-1 inhibitors **20a** and **20b**, antileishmanial agent **20c**, and CF_3_- and CHF_2_-substituted quinolines **20d** and **20e**, shown in Scheme 6 [86,87,88].

**Scheme 7:**
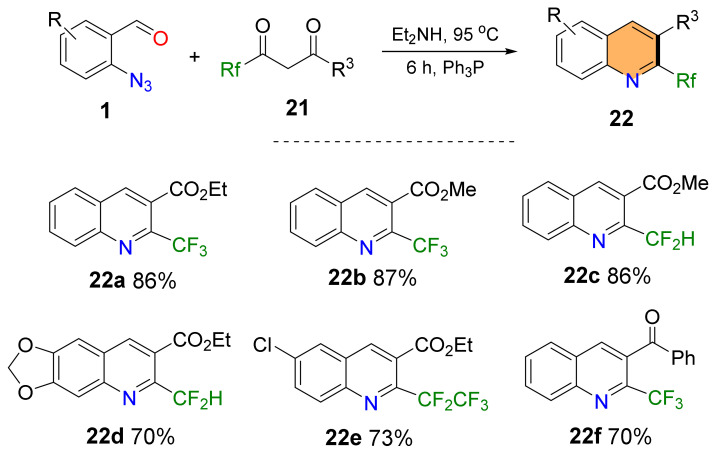
Direct synthesis of 2-fluorinated quinolines **22**.

**Scheme 8:**
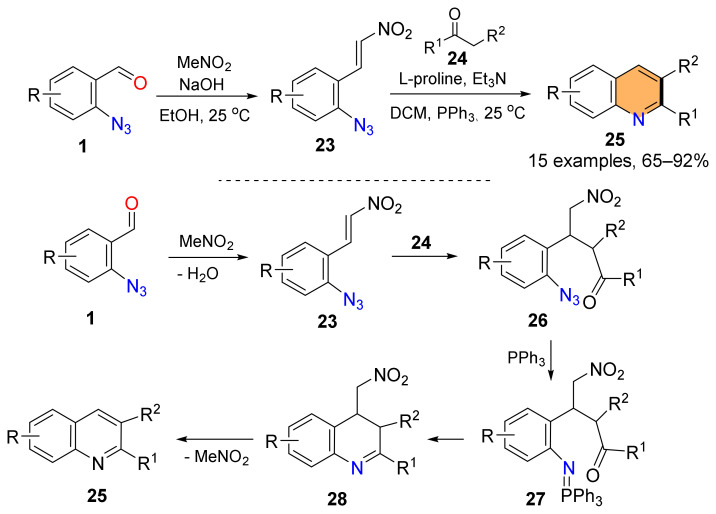
One-pot synthesis of 2,3-substituted quinolines **25**.

Due to the importance of F groups in drug molecules [89,90,91,92,93], the Zhang group introduced a reaction between compounds **1** and **21** for making 2-fluorinated quinolines **22a**–**f**, bearing CF_2_, CF_3_, and C_2_F_5_ at 70–87% yields (Scheme 7). These compounds are related to histone acetyltransferase (HAT) inhibitors [94,95,96]. Shi and co-workers reported the synthesis of 2,3-substituted quinolines **25** via a condensation/Michael/Staudinger/aza-Wittig/aromatization reaction (Scheme 8) [97]. The *ortho*-Azido-*β*-nitro-styrenes **23**, synthesized by the condensation of 2-azidobenzaldehydes **1** and MeNO_2_, undergo the Michael addition with ketones **24** to form **26**, which then reacts with PPh_3_ according to the Staudinger reaction to afford intermediates **27**. The intramolecular aza-Wittig reaction of **27** gives **28**, followed by the aromatic removal of MeNO_2_ to afford **25** (Scheme 8).

**Scheme 9:**
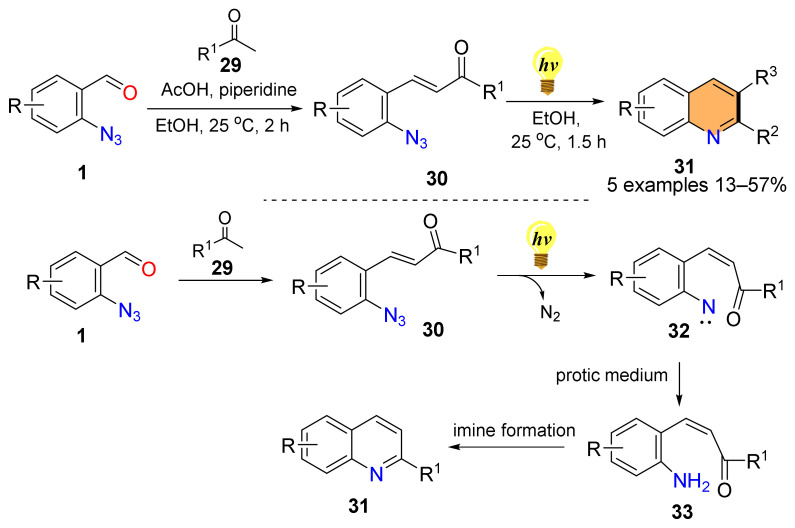
Photosynthesis of 2-substituted quinolines **31**.

In addition to aza-Wittig cyclization, for making quinolines, Chassaing and co-workers reported intramolecular imine formation of aldehyde and aniline for the synthesis of 2-substituted quinolines **31**. The reaction process involves the formation of *ortho*-azidocinnamoyl compounds **30** via the condensation of 2-azidobenzaldehydes **1** and ketones **29**. The irradiation of aryl azides **30** generates nitrenes **32**, which are then converted into amino intermediates **33** in protic media to give products **31** after imine cyclization (Scheme 9) [98].

**Scheme 10:**
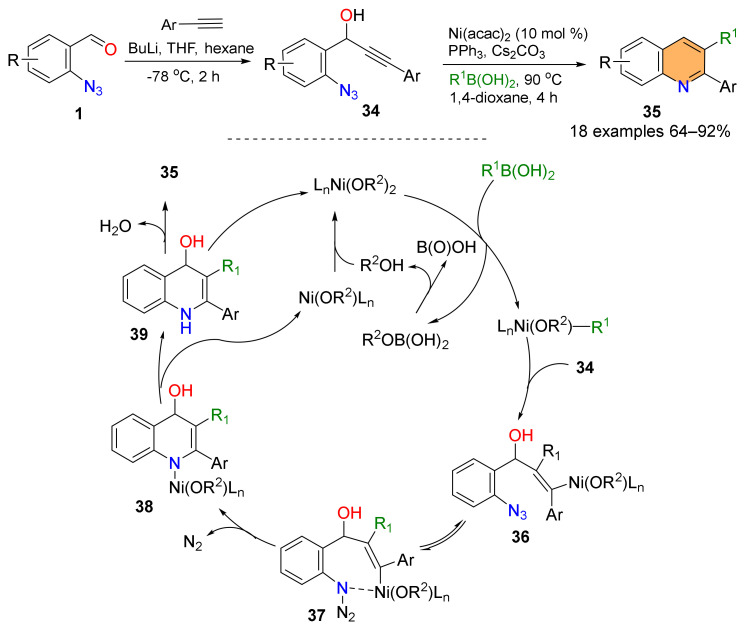
Ni-catalyzed cyclization for making 2,3-substituted quinolines **35**.

Reddy and co-workers reported a cyclization reaction of azidophenyl propargyl alcohol **34** for making 2,3-substituted quinolines **35** (Scheme 10) [99]. The azidophenyl propargyl alcohol **34**, generated according to the Favorskii reaction of 2-azidobenzaldehydes **1**, undergoes the Ni-catalyzed addition of its R^1^ group to the propargylic system to form intermediates **36**, followed by *E*/*Z* isomerization to afford intermediate **37**. The stabilized azido group enables the subsequent denitrogenative cyclization into cyclic intermediates **38**. The denickelation of **38** under the photo conditions gives intermediate **39**, followed by dehydrative aromatization to afford 2,3-substituted quinolines **35**.

**Scheme 11:**
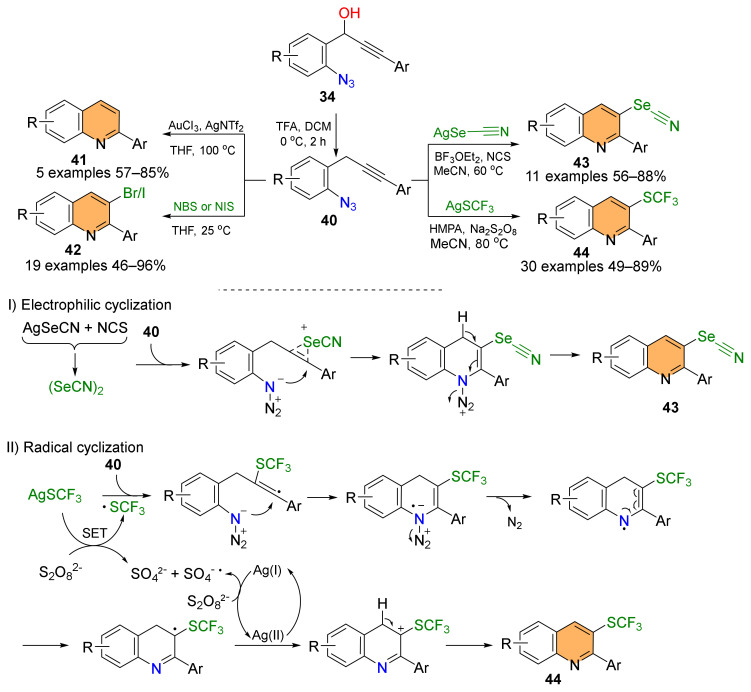
Alkyne–azide cyclization for making quinolines **41**–**44**.

The Yamamoto group utilized *o*–propargyl arylazides **40** derived from the dehydration of compound **34** for the synthesis of multi-substituted quinolines **41**–**44** (Scheme 11). The electrophilic cyclization of **40** in the presence of catalytic amounts of AuCl_3_/AgNTf_2_ affords products **41**. The method was applied to the synthesis of 3-Br/I quinolines **42** in the presence of I_2_, Br_2_, or NIS [100]. Zhou and co-workers extended the scope of electrophilic cyclization induced by pseudohalogen (SeCN)_2_ for the synthesis of quinolylselenocyanates **43** [101]. Wang and Quan reported the synthesis of SCF_3_-substituted quinolines **44** through radical cyclization of *o*–propargyl arylazides **40** [102].

**Scheme 12:**
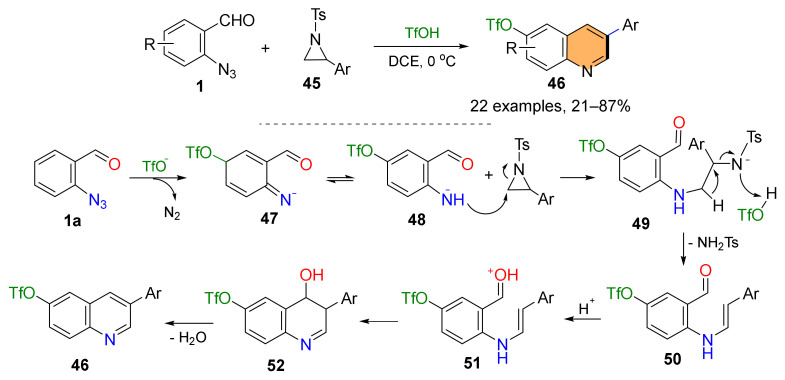
Ring-opening and cyclization of aziridines for the synthesis of quinolines **46**.

The ring-opening of aziridines is a useful tool for the construction of N-heterocycles via cycloaddition or cyclization reactions [103,104,105,106]. Wan and co-workers utilized ring-opening and the cyclization of aziridines **45** for the synthesis of 3-substituted quinolines **46** (Scheme 12) [107]. The TfOH-promoted denitrogenation of the azide group of **1** gave intermediate **47** and then **48** after deprotonation. The addition of 48 to aziridines **45** forms **49**, followed by the cleavage of TsNH2 to afford **50**. Acid-promoted cyclization of **50** gives **51**, which is converted into 3-aryl quinoline **46** after aromatization (Scheme 12).

### 2.2. Synthesis of Polycyclic Quinolines

**Figure 3:**
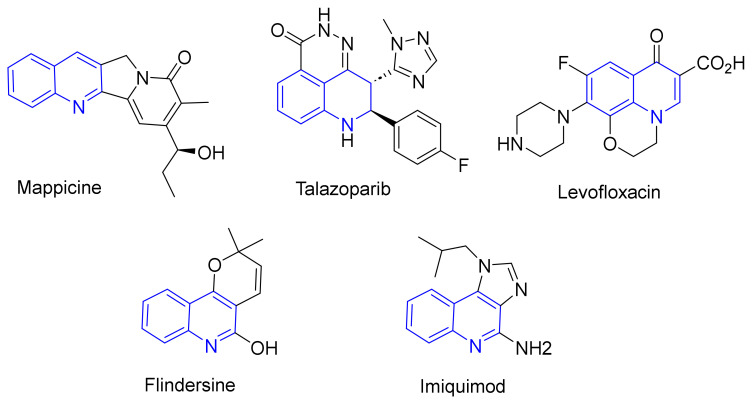
Polycyclic quinoline-based marked drugs and natural products.

**Scheme 13:**
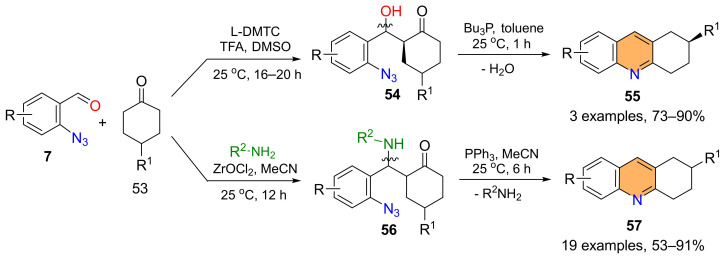
One-pot synthesis of cyclohexane-fused quinolines **55** and **57**.

Polycyclic quinolines can be found in a number of commercial drugs and other bioactive compounds, such as the natural alkaloid Mappicine [108], the poly ADP ribose polymerase (PARP) inhibitor for cancer Talazoparib [109], the antibacterial drug Levofloxacin [110], the natural product Flindersine [111], and the immune response modifier Imiquimod (Figure 3) [112]. The cyclization of 2-azidobenzaldehydes can be integrated into multistep synthesis, one-pot synthesis, or a multi-component reaction (MCR) to make *N*-heterocycles [63,68,113,114]. The aza-Wittig cyclization-based synthesis of polycyclic quinolines is covered in this section. Shown in Scheme 13 are two pathways for the synthesis of cyclohexane-fused quinolines **55** (ee up to 98%) and **57** involving Knoevenage/aza-Wittig/dehydration reactions and Mannich/aza-Wittig/deamination sequences, respectively [115,116]. 

**Scheme 14:**
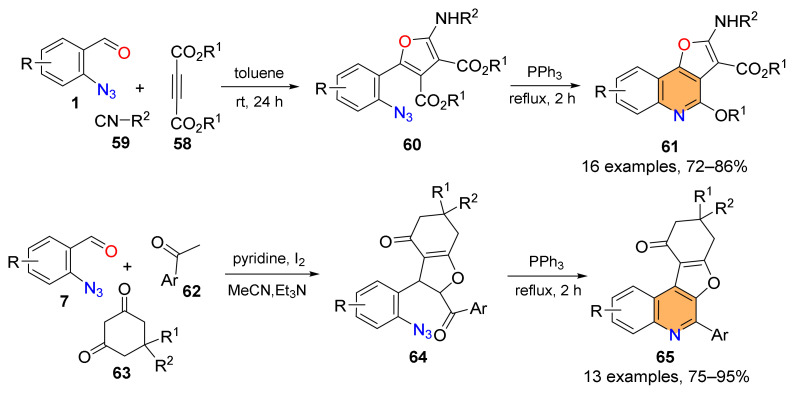
MCR-initiated synthesis of furan-fused quinolines **61** and **65**.

A three-component reaction (3-CR)-initiated synthesis of furan-fused quinolines was reported by Ding and co-workers (Scheme 14). Compounds **60** generated from the condensation of 2-azidobenzaldehydes **1**, dialkyl acetylenedicarboxylate **58**, and isocyanides **59** are used for Staudinger and aza-Wittig cyclization to make furan-fused quinolines **61** [117]. He’s group reported sequential 3-CR/Staudinger/aza-Wittig cyclization/dehydroaromatization reaction processes for making furan-fused quinolines **65** (Scheme 14) [118].

**Scheme 15:**
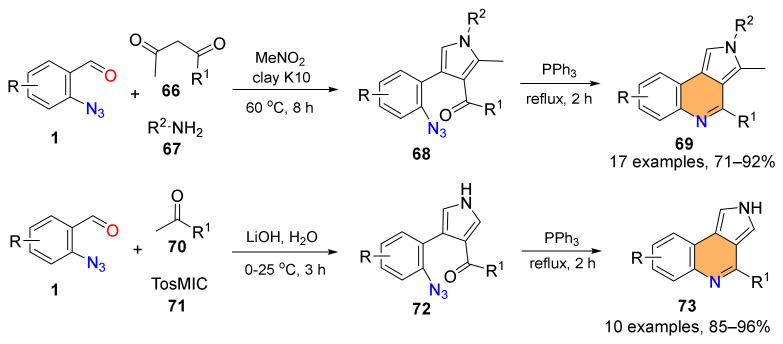
MCR-initiated synthesis of pyrrole-fused quinolines **69** and **73**.

**Scheme 16:**
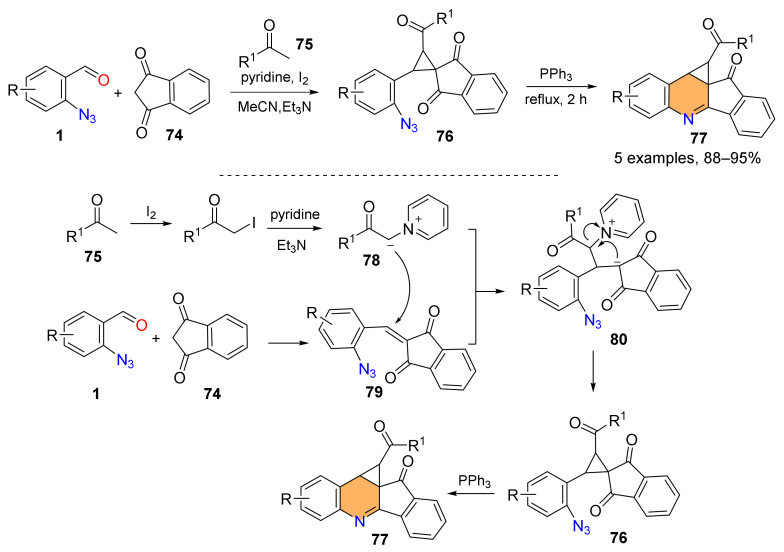
MCR-initiated synthesis of highly condensed quinolines **77**.

He and Bharate’s groups independently reported the reaction of aldehydes, 1,3-dicarbonyl compounds **66**, amines **67**, and nitroalkanes to make pyrrole-fused quinolines **69** (Scheme 15) [119,120]. He and co-workers also reported a 3-CR/Staudinger/aza-Wittig process for making pyrrole-fused quinolines **73** using **1**, acetyl compounds **70**, and TosMIC **71** as the starting materials (Scheme 15) [121]. Interestingly, an example of making cyclopropa[*c*]indeno [1,2-*b*]quinolines **77** was developed according to the 3-CR/Staudinger/aza-Wittig sequence. The products have a highly condensed ring system, including cyclopropane (Scheme 16) [122].

**Scheme 17:**
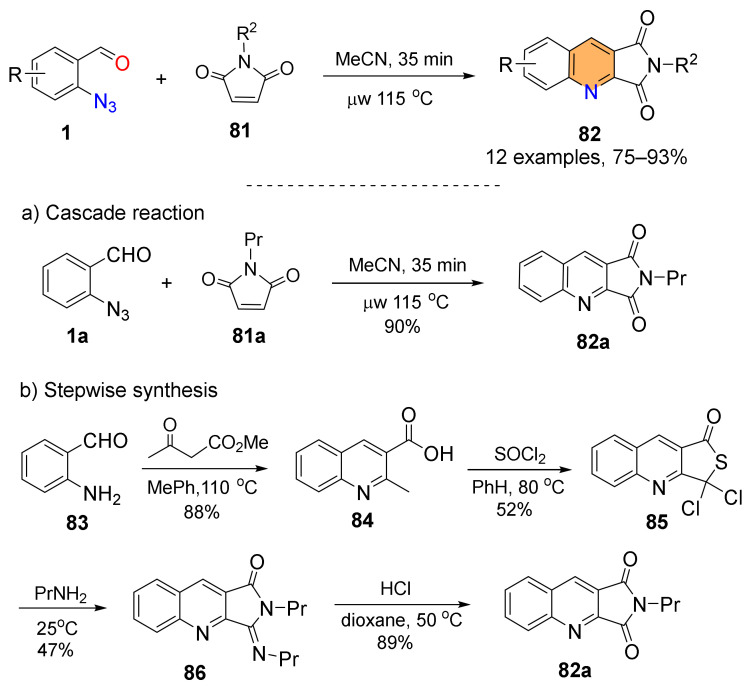
Cascade reactions for pyrroloquinolinediones **82**.

**Scheme 18:**
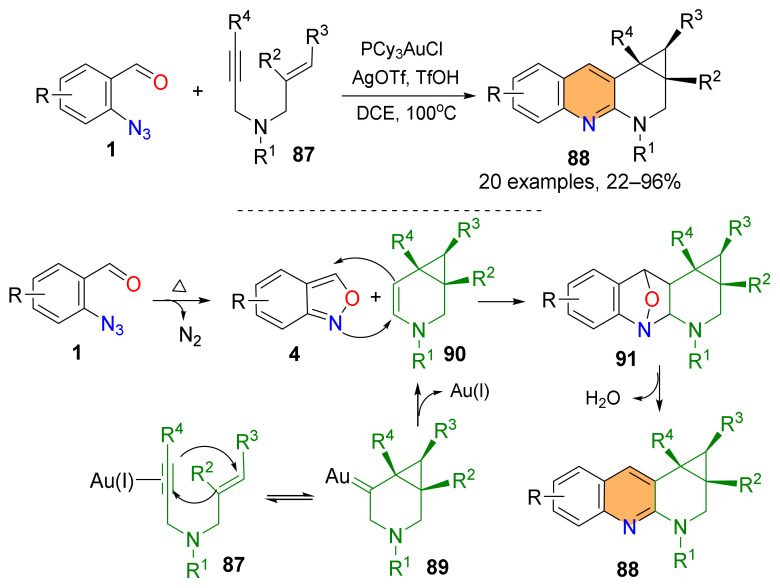
Cascade reactions for tetrahydrobenzo[*b*][1,8]naphthyridines **88**.

Other than aza-Wittig cyclization for the construction of quinolines, Zhang and co-workers reported cascade denitrogenation/aza-Diels–Alder/dehydrative aromatization reactions for the synthesis of pyrrolidine-2,5-dione-fused quinolines using 2-azidobenzaldehydes **1** and *N*-substituted maleimides **81** as the starting materials (Scheme 17) [79]. This method (Scheme 17a) is more efficient than stepwise synthesis for the synthesis of **82a** (Scheme 17b) [123]. In another case, Li and Wang’s group utilized compounds **1** and 3-aza-1,6-enynes **87** as the substrates for making tetrahydrobenzo[*b*][1,8]naphthyridines **88** (Scheme 18) [124]. The reactions involved aza-Diels–Alder cycloaddition of intermediates **90** derived from the Au-catalyzed reaction of enynes **87** to form **91**, followed by dehydrative aromatization to give products **88**.

**Scheme 19:**
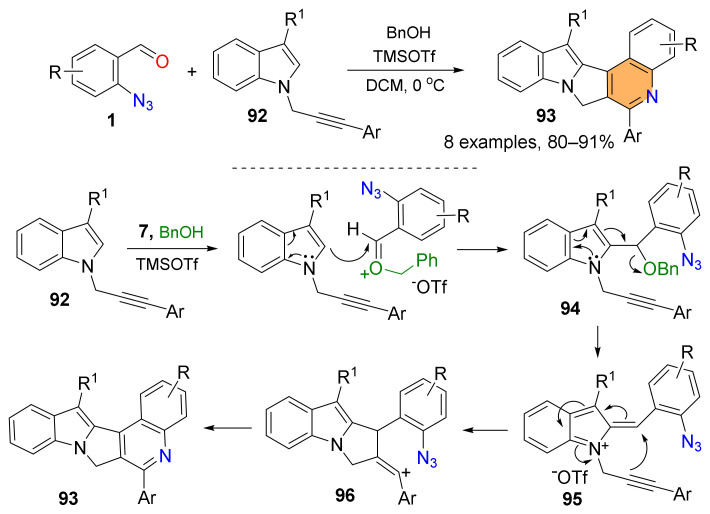
Cascade synthesis of pyrrolizino-quinolines **93**.

**Scheme 20:**
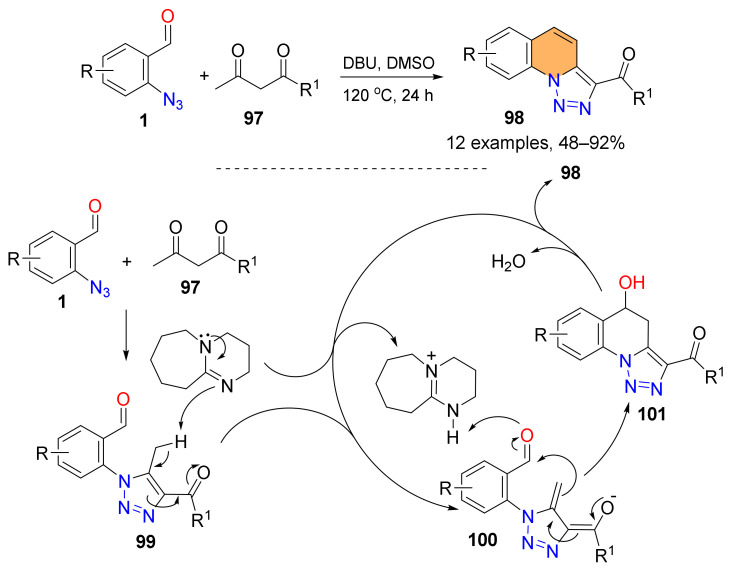
Direct synthesis of [1,2,3] triazolo[1,5-a] quinolines **98**.

Gharpure and co-workers introduced Lewis-acid-promoted cascade reactions involving Friedel–Crafts/alkyne indol-2-yl cation cyclization/vinyl cation trapping for the synthesis of pyrrolizino-quinolines **93** [125]. Benzyl alcohol and TMSOTf promote the Friedel–Crafts reaction of **1** and indoles **92** to form **93**, which undergoes alcohol elimination and cyclization to afford vinyl cations **96**. The trapping of the vinyl cations with the azide group generates pyrrolizino-quinolines **93** (Scheme 19) [125]. Alves and co-workers introduced sequential [3+2] cycloaddition and condensation reactions for making triazole-fused quinolines **98** (Scheme 20) [126]. The 1,3-dipolar cycloaddition of **1** and 1,3-dicarbonyl compounds **97** leads to the formation of 1,2,3-triazoles **99**. The intramolecular condensation of enolates **100** generated from the DBU deprotonation of **99** affords **101** and then triazole-fused quinolines **98** after dehydration.

**Scheme 21:**
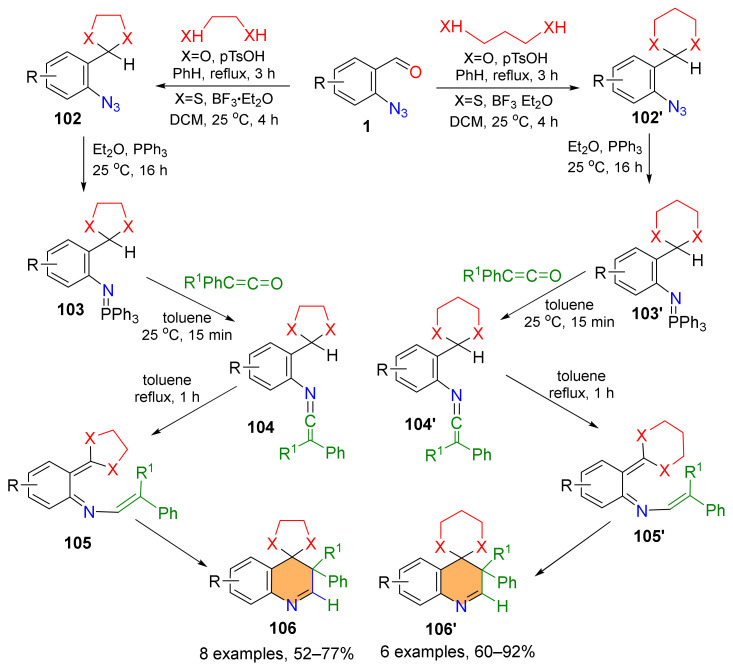
Tandem synthesis of spiroquinolines **106** and **106′**.

Alajarin and co-workers reported the cyclization of ketenimines for making spiroquionlines **106** and **106′** using azides **102** and **102′** bearing five- and six-membered cyclic acetal functions (such as 1,3-dioxolane, 1,3-dithiolane, 1,3-dioxane, and 1,3-dithiane) as the starting materials (Scheme 21) [127,128]. The treatment of azides **102** or **102′** with PPh_3_ affords iminophosphorane **103** or **103′**, followed by aza-Wittig reactions with disubstituted ketenes giving ketenimines **104** or **104′** and then *ortho*-azaxylylenes **105** or **105′** after deprotonation. The cyclization of **105** or **105′** gives the products spiroquinolines **106** or **106′**.

### 2.3. Synthesis of 4-Hydroxyquinoline Derivatives

**Figure 4:**
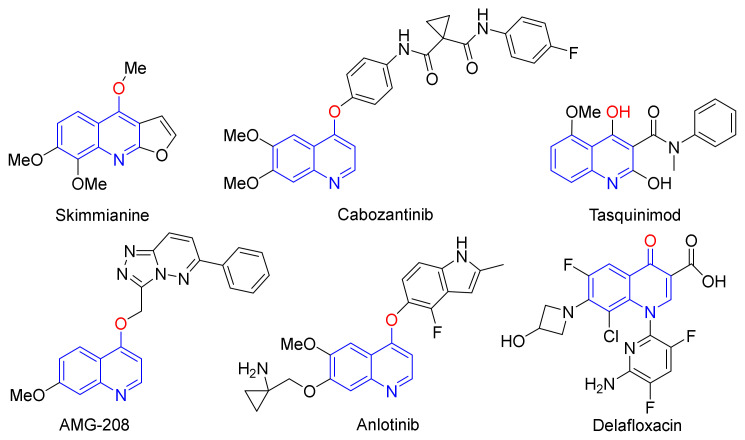
Examples of 4-hydroxy and related quinolines as drug molecules.

There are some drugs and natural products that have 4-hydroxyquinoline moiety, such as the furoquinoline alkaloid Skimmianine, with anticancer and anti-inflammatory effects [129,130]; Cabozantinib, used for the treatment of medullary thyroid cancer [131]; Tasquinimod as an immunomodulator for the treatment of blood cancers [132]; AMG-208 as a clinical trial drug for cancer [133]; Anlotinib, with antineoplastic and anti-angiogenic activities [134,135]; and Delafloxacin as a fluoroquinolone antibiotic for the treatment of acute bacterial skin and skin structure infections (Figure 4) [136].

**Scheme 22:**
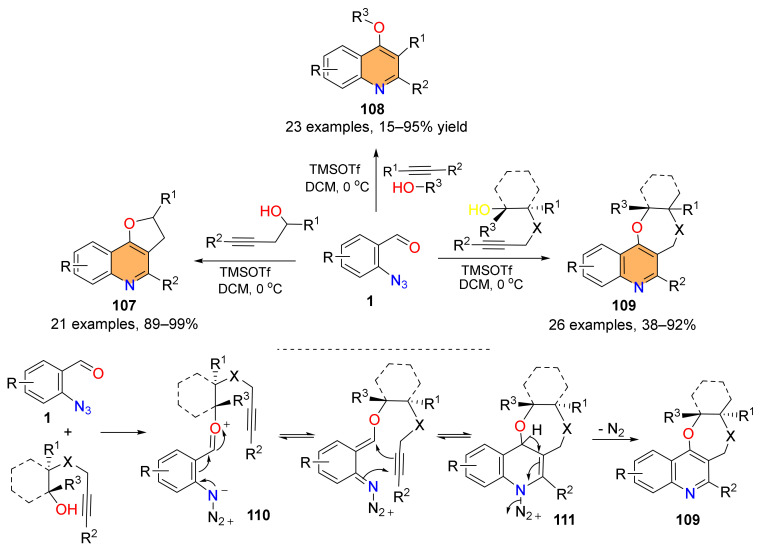
Cascade synthesis of 4-alkoxy quinolines **107**, **108**, and **109**.

Other than aniline-based [3+3] annulation [137] and [4+2] annulation [138] reactions for making 4-hydroxyquinolines, 2-azidobenzaldehydes have also been employed for the [4+2] annulation-based synthesis of 4-alkoxy quinolines. Gharpure and coworkers reported the reactions of **1** with hydroxyalkynes for the synthesis of different kinds of 4-alkoxy quinolines, **107**, **108**, and **109** (Scheme 22) [139,140,141]. The synthesis first led to the formation of oxonium ions **110** followed by intramolecular [4+2] cycloaddition and aromatization to give 4-alkoxy quinolines **109**, with a new seven-membered heterocyclic ring (Scheme 22).

**Scheme 23:**
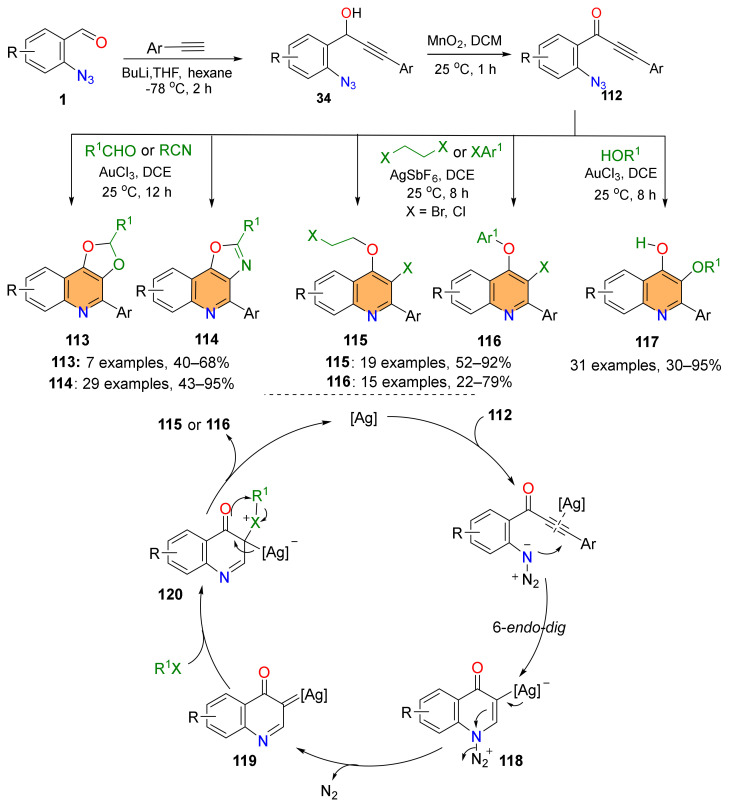
Metal-catalyzed synthesis of diverse 4-alkoxy quinolines **113**–**117**.

Metal-catalyzed 6-*endo*-dig azide–yne cyclization of azide-tethered alkynes **112** has been developed for the synthesis of multi-substituted 4-alkoxy quinolines **113**–**117** (Scheme 23) [142,143,144]. For example, Xu and coworkers synthesized **115** and **116** via the AgSbF_6_-catalyzed cyclization of azide-tethered alkynes. The cyclization of Ag(I)-activated alkyne-tethered azides **112** affords **118**, followed by N_2_ gas release from *α*-imino silver carbenes **119** and then reaction with R^1^X to afford halonium zwitterions **120**. After a concerted rearrangement of **120**, product **115** or **116** is obtained.

**Scheme 24:**
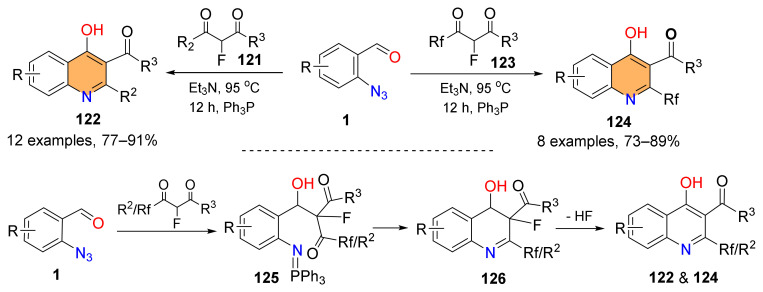
One-pot synthesis of quinolin-4-ols **122** and **124**.

**Scheme 25:**
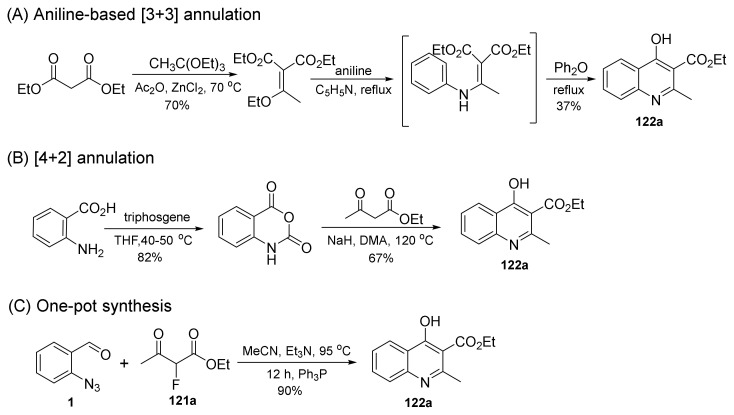
Three methods for making **122a**.

Zhang and coworkers reported the direct synthesis of quinolin-4-ols, which are structurally related to histone acetyltransferase (HAT) inhibitors [145] and the key intermediates for making HMG-CoA reductase inhibitors [146]. The reaction of **1** and *α*-fluoro-*β*-ketoesters **121** or **123** via sequential aldol, aza-Wittig, and dehydrofluorinative aromatization reactions gives quinolin-4-ols **122** or **124** (Scheme 24) [83]. Green chemistry metrics analysis for this one-pot synthesis and two reported multi-step syntheses [138] of HAT inhibitor **144a** was conducted [79,82,147] to validate synthetic efficiency and low waste generation (Scheme 25).

### 2.4. Synthesis of 4-Amino-Quinolines

**Figure 5:**
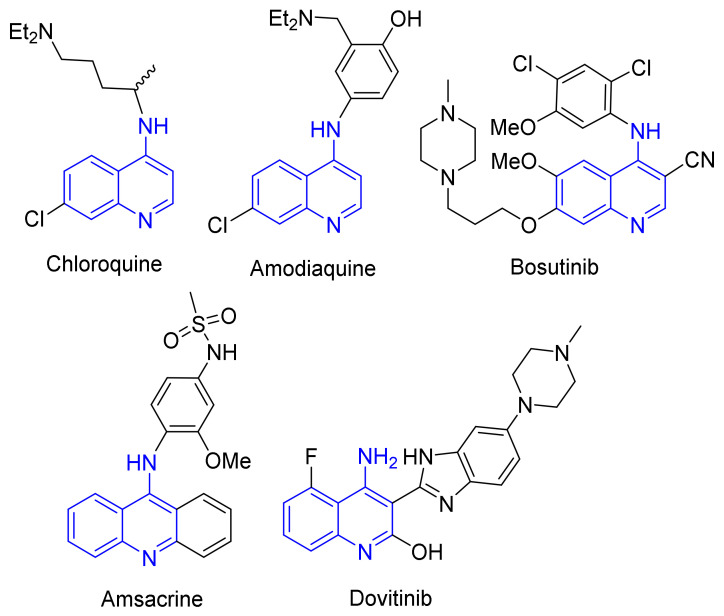
4-Aminoquinoline-based drugs and drug candidates.

**Scheme 26:**
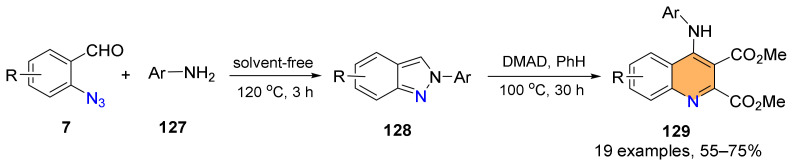
One-pot synthesis of 4-aminoquinolines **129**.

**Scheme 27:**
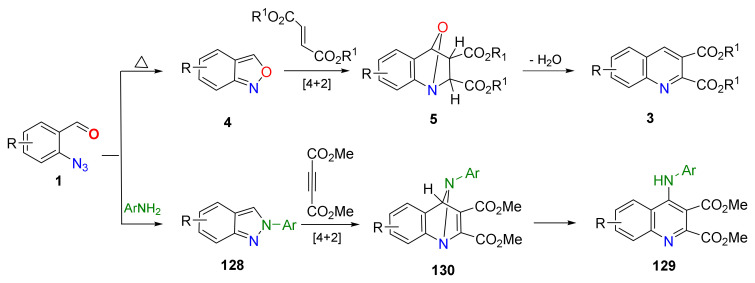
Comparison of [4+2] cycloaddition for making compounds **3** and **129**.

Shown in Figure 5 are some 4-amino-quinoline drugs and drug candidates such as Chloroquine, Amodiaquine, Bosutinib, Amsacrine, and Dovitinib for the treatment of malarial [148,149,150], leukemia [151,152], and cancer diseases [153]. Common methods in the literature for the synthesis of 4-aminoquinolines are aniline-based multi-step reactions [96,154,155,156]. Sharada and coworkers employed azides **1** and amines **127** for the synthesis of 4-aminoquinolines **129** through the aza-Diels–Alder reaction of intermediates **128** with dimethylacetylenedicarboxylate (DMAD) (Scheme 26) [157]. Shown in Scheme 27 are two examples of quinoline synthesis using 2-azidobenzaldehyde-based [4+2] cycloadditions. In the first case of using fumarate esters as the dienophiles, quinolines **3** are generated from dehydrative aromatization involving C-O bond cleavage. In the second case of using DMAD as a dienophile for cycloaddition with **128**, 4-aminoquinolines **129** result from the aromatization via the N-N bond cleavage of **130** [79,157].

**Scheme 28:**
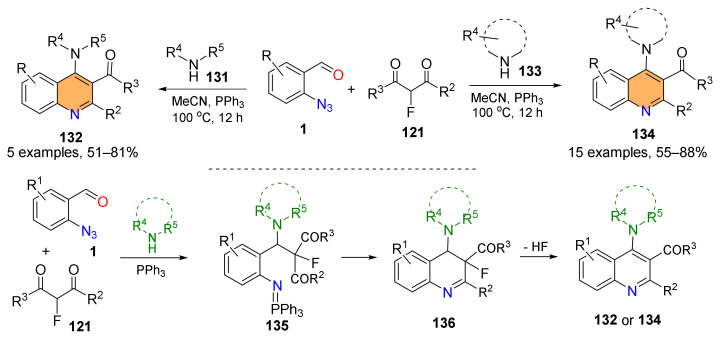
Three-component reaction to make 4-aminoquinolines **132** and **134**.

**Scheme 29:**
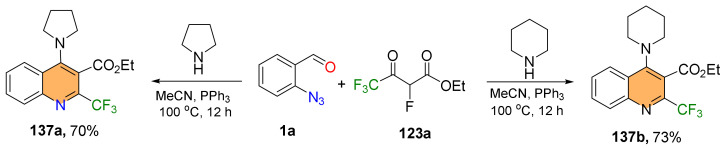
One-pot synthesis of CF_3_-containing 4-aminoquinolines **137a** and **137b**.

Zhang and co-workers reported a three-component reaction of azides **1**, *α*-fluoro-*β*-ketoesters **121**, and amines **131** or **133** for the synthesis of 4-aminoquinolines **132** and **134** involving Mannich, aza-Wittig and dehydrofluorinative aromatization reactions (Scheme 28) [158]. They also applied this method to the synthesis of 2-CF_3_ quinolines **137a** and **137b** with a pyrrolidine or a piperidine at the 4-position (Scheme 29).

## 3. Conclusions

Presented in this paper are 2-azidobenzaldehyde-initiated reactions for the synthesis of diverse quinoline compounds, including fused, spiro-, and polycyclic quinolines, as well as substituted quinolines such as quinoline-4-ols and 4-aminoquinolines. These biologically significant quinoline compounds could be well utilized in medicinal chemistry programs for drug discovery. Also, 2-azidobenzaldehyde-initiated synthesis can be developed as one-pot stepwise synthesis or a multicomponent reaction for operation simplicity, process efficiency, and economizing in terms of steps, pots, and atoms. Some of the synthetic methods presented in this paper provide novel pathways for making quinolines which could also be used for the synthesis of other heterocyclic compounds.

## References

[1] Chung P.Y., Bian Z.X.H., Pun Y.D., Chan A.S., Chui C.H., Tang J.C., Lam K.H. (2015). Recent advances in research of natural and synthetic bioactive quinolines. Future Med. Chem..

[2] Kumar S., Bawa S., Gupta H. (2009). Biological activities of quinoline derivatives. Mini Rev. Med. Chem..

[3] Broch S., Henon H., Debaud L.A., Fogeron M.L., Bonnefoy-Berard N., Anizon F., Moreau P. (2010). Synthesis and biological activities of new di- and trimeric quinoline derivatives. Bioorg. Med. Chem..

[4] Dib M., Ouchetto H., Ouchetto K., Hafid A., Khouili M. (2021). Recent Developments of Quinoline Derivatives and their Potential Biological Activities. Curr. Org. Synth..

[5] Chu X.M., Wang C., Liu W., Liang L.L., Gong K.K., Zhao C.Y., Sun K.L. (2019). Quinoline and quinolone dimers and their biological activities: An overview. Eur. Med. Chem..

[6] Matada B.S., Pattanashettar R., Yernale N.G. (2021). A comprehensive review on the biological interest of quinoline and its derivatives. Bioorg. Med. Chem..

[7] Martins A.C., Paoliello M.M.B., Docea A.O., Santamaria A., Tinkov A.A., Skalny A.V., Aschner M. (2021). Review of the mechanism underlying mefloquine-induced neurotoxicity. Crit. Rev. Toxicol..

[8] Kucharski D.J., Jaszczak M.K., Boratynski P.J. (2022). A Review of Modifications of Quinoline Antimalarials: Mefloquine and (hydroxy)Chloroquine. Molecules.

[9] Dos Santos M.C., Scaini J.L.R., Lopes M.V.C., Rodrigues B.G., Silva N.O., Borges C.R.L., Dos Santos S.C., Dos Santos Machado K., Werhli A.V., da Silva P.E.A. (2021). Mefloquine synergism with anti-tuberculosis drugs and correlation to membrane effects: Biologic, spectroscopic and molecular dynamics simulations studies. Bioorg. Chem..

[10] Cifci B., Yildiz Y., Altin E., Habibi H., Kocer B., Dizbay M. (2023). Successful treatment of HIV-associated progressive multifocal leukoencephalopathy (PML) with mirtazapine, mefloquine, and IVIG combination therapy: A case report. J. Neurovirol..

[11] Dodion P.F., Wagener T., Stoter G., Drozd A., Lev L.M., Skovsgaard T., Renard J., Cavalli F. (1990). Phase II trial with Brequinar (DUP-785, NSC 368390) in patients with metastatic colorectal cancer: A study of the Early Clinical Trials Group of the EORTC. Ann. Oncol..

[12] Andersen P.I., Krpina K., Ianevski A., Shtaida N., Yang E., Jo J., Koit S., Tenson T., Hukkanen V., Anthonsen M.W. (2019). Novel Antiviral Activities of Obatoclax, Emetine, Niclosamide, Brequinar, and Homoharringtonine. Viruses.

[13] Horvat N.K., Lesinski G.B. (2022). Bring on the brequinar: An approach to enforce the differentiation of myeloid-derived suppressor cells. J. Clin. Investig..

[14] Sadeq A., Elnour A.A., Farah F.H., Ramadan A., Baraka M.A., Don J., Amoodi A.A., Sam K.G., Mazrouei N.A., Alkaabi M. (2023). A Systematic Review of Randomized Clinical Trials on the Efficacy and Safety of Pitavastatin. Curr. Rev. Clin. Exp. Pharmacol..

[15] Katanasaka Y., Hirano S., Sunagawa Y., Miyazaki Y., Sato H., Funamoto M., Shimizu K., Shimizu S., Sari N., Hasegawa K. (2021). Clinically Administered Doses of Pitavastatin and Rosuvastatin. Int. Heart J..

[16] Gowripattapu S., Sathis Kumar D., Selvamuthukumar S. (2023). Formulation and Statistical Evaluation of Tablets Containing Pitavastatin- Self Nano Emulsifying Drug Delivery Systems. Curr. Drug Deliv..

[17] Weisinger H.S., Pesudovs K., Collin H.B. (2000). Management of patients undergoing hydroxychloroquine (Plaquenil) therapy. Clin. Exp. Optom..

[18] Veld A.E.I., Grievink H.W., van der Plas J.L., Eveleens Maarse B.C., van Kraaij S.J.W., Woutman T.D., Schoonakker M., Klarenbeek N.B., de Kam M.L., Kamerling I.M.C. (2023). Immunosuppression by hydroxychloroquine: Mechanistic proof in in vitro experiments but limited systemic activity in a randomized placebo-controlled clinical pharmacology study. Immunol. Res..

[19] Chen Z., Wu J., Wang W., Tang X., Zhou L., Lv Y., Zheng Y. (2023). Investigation of the Pathogenic Mechanism of Ciprofloxacin in Aortic Aneurysm and Dissection by an Integrated Proteomics and Network Pharmacology Strategy. J. Clin. Med..

[20] Loupias P., Laumaille P., Morandat S., Mondange L., Guillier S., El Kirat K., Da Nascimento S., Biot F., Taudon N., Dassonville-Klimpt A. (2023). Synthesis and study of new siderophore analog-ciprofloxacin conjugates with antibiotic activities against *Pseudomonas aeruginosa* and *Burkholderia* spp.. Eur. J. Med. Chem..

[21] Yuan L., Wu H., Wang J., Zhou M., Zhang L., Xiang J., Liao Q., Luo L., Qian M., Zhang D. (2023). Pharmacokinetics, withdrawal time, and dietary risk assessment of enrofloxacin and its metabolite ciprofloxacin, and sulfachloropyridazine-trimethoprim in Taihe black-boned silky fowls. J. Food Sci..

[22] Bo Z., Chen B., Zhao Z., He Q., Mao Y., Yang Y., Yao F., Yang Y., Chen Z., Yang J. (2023). Prediction of Response to Lenvatinib Monotherapy for Unresectable Hepatocellular Carcinoma by Machine Learning Radiomics: A Multicenter Cohort Study. Clin. Cancer Res..

[23] Peng Z., Fan W., Zhu B., Wang G., Sun J., Xiao C., Huang F., Tang R., Cheng Y., Huang Z. (2023). Lenvatinib Combined with Transarterial Chemoembolization as First-Line Treatment for Advanced Hepatocellular Carcinoma: A Phase III, Randomized Clinical Trial (LAUNCH). J. Clin. Oncol..

[24] Wang X.H., Liu C.J., Wen H.Q., Duan X.H., Jiao Y.Q., Liu Y.J., Chen M.S., Zhu K.S., Mao X.H., Zhou Q.F. (2023). Effectiveness of lenvatinib plus immune checkpoint inhibitors in primary advanced hepatocellular carcinoma beyond oligometastasis. Clin. Transl. Med..

[25] Afzal O., Kumar S., Haider M.R., Ali M.R., Kumar R., Jaggi M., Bawa S. (2015). A review on anticancer potential of bioactive heterocycle quinoline. Eur. J. Med. Chem..

[26] Arafa R.K., Hegazy G.H., Piazza G.A., Abadi A.H. (2013). Synthesis and in vitro antiproliferative effect of novel quinoline-based potential anticancer agents. Eur. J. Med. Chem..

[27] Fu H.G., Li Z.W., Hu X.X., Si S.Y., You X.F., Tang S., Wang Y.X., Song D.Q. (2019). Synthesis and Biological Evaluation of Quinoline Derivatives as a Novel Class of Broad-Spectrum Antibacterial Agents. Molecules.

[28] Dorababu A. (2021). Recent update on antibacterial and antifungal activity of quinoline scaffolds. Arch. Pharm..

[29] Jin G., Xiao F., Li Z., Qi X., Zhao L., Sun X. (2020). Design, Synthesis, and Dual Evaluation of Quinoline and Quinolinium Iodide Salt Derivatives as Potential Anticancer and Antibacterial Agents. ChemMedChem.

[30] Chen Y.J., Ma K.Y., Du S.S., Zhang Z.J., Wu T.L., Sun Y., Liu Y.Q., Yin X.D., Zhou R., Yan Y.F. (2021). Antifungal Exploration of Quinoline Derivatives against Phytopathogenic Fungi Inspired by Quinine Alkaloids. J. Agric. Food Chem..

[31] Cretton S., Dorsaz S., Azzollini A., Favre-Godal Q., Marcourt L., Ebrahimi S.N., Voinesco F., Michellod E., Sanglard D., Gindro K. (2016). Antifungal Quinoline Alkaloids from Waltheria indica. J. Nat. Prod..

[32] Yang G.Z., Zhu J.K., Yin X.D., Yan Y.F., Wang Y.L., Shang X.F., Liu Y.Q., Zhao Z.M., Peng J.W., Liu H. (2019). Design, Synthesis, and Antifungal Evaluation of Novel Quinoline Derivatives Inspired from Natural Quinine Alkaloids. J. Agric. Food Chem..

[33] Das P., Deng X., Zhang L., Roth M.G., Fontoura B.M., Phillips M.A., De Brabander J.K. (2013). SAR-Based Optimization of a 4-Quinoline Carboxylic Acid Analogue with Potent Antiviral Activity. ACS Med. Chem. Lett..

[34] De la Guardia C., Stephens D.E., Dang H.T., Quijada M., Larionov O.V., Lleonart R. (2018). Antiviral Activity of Novel Quinoline Derivatives against Dengue Virus Serotype 2. Molecules.

[35] Seliem I.A., Panda S.S., Girgis A.S., Moatasim Y., Kandeil A., Mostafa A., Ali M.A., Nossier E.S., Rasslan F., Srour A.M. (2021). New quinoline-triazole conjugates: Synthesis, and antiviral properties against SARS-CoV-2. Bioorg. Chem..

[36] Wang M., Zhang G., Zhao J., Cheng N., Wang Y., Fu Y., Zheng Y., Wang J., Zhu M., Cen S. (2021). Synthesis and antiviral activity of a series of novel quinoline derivatives as anti-RSV or anti-IAV agents. Eur. J. Med. Chem..

[37] Vinindwa B., Dziwornu G.A., Masamba W. (2021). Synthesis and Evaluation of Chalcone-Quinoline Based Molecular Hybrids as Potential Anti-Malarial Agents. Molecules.

[38] Roy D., Anas M., Manhas A., Saha S., Kumar N., Panda G. (2022). Synthesis, biological evaluation, Structure—Activity relationship studies of quinoline-imidazole derivatives as potent antimalarial agents. Bioorg. Chem..

[39] Jesu Jaya Sudan R., Lesitha Jeeva Kumari J., Iniyavan P., Sarveswari S., Vijayakumar V. (2023). Evaluation of xanthene-appended quinoline hybrids as potential leads against antimalarial drug targets. Mol. Divers..

[40] Orhan Puskullu M., Tekiner B., Suzen S. (2013). Recent Studies of Antioxidant Quinoline Derivatives. Mini Rev. Med. Chem..

[41] Parameswaran K., Sivaguru P., Lalitha A. (2013). Synthesis of novel bis(pyrimido [5,4-c]quinoline-2,4(1H,3H)-dione) and its derivatives: Evaluation of their antioxidant properties. Bioorg. Med. Chem. Lett..

[42] Ismaili L., Nadaradjane A., Nicod L., Guyon C., Xicluna A., Robert J.F., Refouvelet B. (2008). Synthesis and antioxidant activity evaluation of new hexahydropyrimido [5,4-c]quinoline-2,5-diones and 2-thioxohexahydropyrimido [5,4-c]quinoline-5-ones obtained by Biginelli reaction in two steps. Eur. J. Med. Chem..

[43] Tseng C.H., Tung C.W., Wu C.H., Tzeng C.C., Chen Y.H., Hwang T.L., Chen Y.L. (2017). Discovery of Indeno [1,2-c]quinoline Derivatives as Potent Dual Antituberculosis and Anti-Inflammatory Agents. Molecules.

[44] El-Feky S.A., Abd El-Samii Z.K., Osman N.A., Lashine J., Kamel M.A., Thabet H. (2015). Synthesis, molecular docking and anti-inflammatory screening of novel quinoline incorporated pyrazole derivatives using the Pfitzinger reaction II. Bioorg. Chem..

[45] Chia E.W., Pearce A.N., Berridge M.V., Larsen L., Perry N.B., Sansom C.E., Godfrey C.A., Hanton L.R., Lu G.L., Walton M. (2008). Synthesis and anti-inflammatory structure–activity relationships of thiazine–quinoline–quinones: Inhibitors of the neutrophil respiratory burst in a model of acute gouty arthritis. Bioorg. Med. Chem..

[46] Bewley B.R., Spearing P.K., Weiner R.L., Luscombe V.B., Zhan X., Chang S., Cho H.P., Rodriguez A.L., Niswender C.M., Conn P.J. (2017). Discovery of a novel, CNS penetrant M4 PAM chemotype based on a 6-fluoro-4-(piperidin-1-yl)quinoline-3-carbonitrile core. Bioorg. Med. Chem. Lett..

[47] Zajdel P., Marciniec K., Maslankiewicz A., Satala G., Duszynska B., Bojarski A.J., Partyka A., Jastrzebska-Wiesek M., Wrobel D., Wesolowska A. (2012). Quinoline- and isoquinoline-sulfonamide derivatives of LCAP as potent CNS multi-receptor—5-HT1A/5-HT2A/5-HT7 and D2/D3/D4—Agents: The synthesis and pharmacological evaluation. Bioorg. Med. Chem..

[48] Kumar S., Kaushik D., Bawa S., Khan S.A. (2012). Design, Synthesis and Screening of Quinoline-Incorporated Thiadiazole as a Potential Anticonvulsant. Chem. Biol. Drug Des..

[49] Muruganantham N., Sivakumar R., Anbalagan N., Gunasekaran V., Leonard J.T. (2004). Synthesis, Anticonvulsant and Antihypertensive Activities of 8-Substituted Quinoline Derivatives. Biol. Pharm. Bull..

[50] Shanley H.T., Taki A.C., Byrne J.J., Jabbar A., Wells T.N.C., Samby K., Boag P.R., Nguyen N., Sleebs B.E., Gasser R.B. (2022). A High-Throughput Phenotypic Screen of the ‘Pandemic Response Box’ Identifies a Quinoline Derivative with Significant Anthelmintic Activity. Pharmaceuticals.

[51] Williams A., Villamor L., Fussell J., Loveless R., Smeyne D., Philp J., Shaikh A., Sittaramane V. (2022). Discovery of Quinoline-Derived Trifluoromethyl Alcohols as Antiepileptic and Analgesic Agents That Block Sodium Channels. ChemMedChem.

[52] Kouznetsov V.V., Mendez L.Y.V., Gomez C.M.M. (2005). Recent Progress in the Synthesis of Quinolines. Curr. Org. Chem..

[53] Weyesa A., Mulugeta E. (2020). Recent advances in the synthesis of biologically and pharmaceutically active quinoline and its analogues: A review. RSC Adv..

[54] Nainwal L.M., Tasneem S., Akhtar W., Verma G., Khan M.F., Parvez S., Shaquiquzzaman M., Akhter M., Alam M.M. (2019). Green recipes to quinoline: A review. Eur. J. Med. Chem..

[55] Prajapati S.M., Patel K.D., Vekariya R.H., Panchal S.N., Patel H.D. (2014). Recent advances in the synthesis of quinolines: A review. RSC Adv..

[56] Barluenga J., Rodriguez F., Fananas F.J. (2009). Recent Advances in the Synthesis of Indole and Quinoline Derivatives through Cascade Reactions. Chem. Asian J..

[57] Ramann G.A., Cowen B.J. (2016). Recent Advances in Metal-Free Quinoline Synthesis. Molecules.

[58] Bharate J.B., Vishwakarma R.A., Bharate J.B. (2015). Metal-free domino one-pot protocols for quinoline synthesis. RSC Adv..

[59] Teja C., Khan F.R.N. (2020). Radical Transformations towards the Synthesis of Quinoline: A Review. Chem. Asian J..

[60] Vessally E., Edjlali L., Hosseinian A., Bekhradnia A., Esrafili M.D. (2016). Novel routes to quinoline derivatives from *N*-propargylamines. RSC Adv..

[61] Doraghi F., Karimian S., Aledavoud S.P., Amini A., Larijani B., Mahdavi M. (2023). 2-azidobenzaldehyde: A versatile scaffold for the generation of *N*-heterocyclic compounds. J. Mol. Struct..

[62] Das S.K., Roy S., Khatua H., Chattopadhyay B. (2018). Ir-Catalyzed Intramolecular Transannulation/C(sp2)–H Amination of 1,2,3,4-Tetrazoles by Electrocyclization. J. Am. Chem. Soc..

[63] Roy S., Das S.K., Khatua H., Das S., Singh K.N., Chattopadhyay B. (2021). Iron-Catalyzed Radical Activation Mechanism for Denitrogenative Rearrangement Over C(sp3)–H Amination. Angew. Chem. Int. Ed..

[64] Ansari A.J., Joshi G., Yadav U.P., Maurya A.K., Agnihotri V.K., Kalra S., Kumar R., Singh S., Sawant D.M. (2019). Exploration of Pd-catalysed four-component tandem reaction for one-pot assembly of pyrazolo [1,5-c]quinazolines as potential EGFR inhibitors. Bioorg. Chem..

[65] Zhao L., Yang M.L., Liu M., Ding M.W. (2022). New efficient synthesis of polysubstituted 3,4-dihydroquinazolines and 4H-3,1-benzothiazines through a Passerini/Staudinger/aza-Wittig/addition/nucleophilic substitution sequence. Beilstein J. Org. Chem..

[66] Vroemans R., Bamba F., Winters J., Thomas J., Jacobs J., Van Meervelt L., John J., Dehaen W. (2018). Sequential Ugi reaction/base-induced ring closing/IAAC protocol toward triazolobenzodiazepine-fused diketopiperazines and hydantoins. Beilstein J. Org. Chem..

[67] Vidyacharan S., Murugan A., Sharada D.S. (2016). C(sp2)–H Functionalization of 2H-Indazoles at C3-Position via Palladium(II)-Catalyzed Isocyanide Insertion Strategy Leading to Diverse Heterocycles. J. Org. Chem..

[68] De Moliner F., Bigatti M., De Rosa C., Banfi L., Riva R., Basso A. (2014). Synthesis of triazolo-fused benzoxazepines and benzoxazepinones via Passerini reactions followed by 1,3-dipolar cycloadditions. Mol. Divers..

[69] Singh B. (2023). Bedaquiline in Drug-Resistant Tuberculosis: A Mini-Review. Curr. Mol. Pharmacol..

[70] Conradie F., Bagdasaryan T.R., Borisov S., Howell P., Mikiashvili L., Ngubane N., Samoilova A., Skornykova S., Tudor E., Variava E. (2022). Bedaquiline–Pretomanid–Linezolid Regimens for Drug-Resistant Tuberculosis. N. Engl. J. Med..

[71] Frampton J.E. (2014). QVA149 (Indacaterol/Glycopyrronium Fixed-Dose Combination): A Review of Its Use in Patients with Chronic Obstructive Pulmonary Disease. Drugs.

[72] Cazzola M., Bardaro F., Stirpe E. (2013). The role of indacaterol for chronic obstructive pulmonary disease (COPD). J. Thorac. Dis..

[73] Umezaki Y., Motomura H., Egashira R., Toyofuku A., Naito T. (2023). A Case of Oral Cenesthopathy Treated with the Combination of Brexpiprazole and Mirtazapine. Clin. Neuropharmacol..

[74] Shapovalov V., Kopanitsa L., Pruteanu L.L., Ladds G., Bailey D.S. (2021). Transcriptomics-Based Phenotypic Screening Supports Drug Discovery in Human Glioblastoma Cells. Cancers.

[75] Murray V., Chen J.K., Galea A.M. (2014). The Potential of Acridine Carboxamide Pt Complexes as Anti-Cancer Agents: A Review. Anti-Cancer Agent. Med. Chem..

[76] Nair V., Menon R.S., Biju A.T., Abhilash K.G. (2012). 1,2-Benzoquinones in Diels–Alder reactions, dipolar cycloadditions, nucleophilic additions, multicomponent reactions and more. Chem. Soc. Rev..

[77] Bharate S.B., Mudududdla R., Bharate J.B., Battini N., Battula S., Yadav R.R., Singh B., Vishwakarma R.A. (2012). Tandem one-pot synthesis of flavans by recyclable silica–HClO_4_ catalyzed Knoevenagel condensation and [4+2]-Diels–Alder cycloaddition. Org. Biomol. Chem..

[78] Houk K.N., Liu F., Yang Z., Seeman J.I. (2021). Evolution of the Diels–Alder Reaction Mechanism since the 1930s: Woodward, Houk with Woodward, and the Influence of Computational Chemistry on Understanding Cycloadditions. Angew. Chem..

[79] Zhang X.F., Dhawan G., Muthengi A., Liu S., Wang W., Legris M., Zhang W. (2017). One-pot and catalyst-free synthesis of pyrroloquinolinediones and quinolinedicarboxylates. Green Chem..

[80] Zheng L., Zeng Z.G., Yan Q., Jia F.C., Jia L., Chen Y. (2018). Copper-Catalyzed Synthesis of 3-NO2 Quinolines from o-Azidobenzaldehyde and Nitro-olefins and its Application in the Concise Synthesis of Quindolines. Adv. Synth. Catal..

[81] Ma X., Zhang X., Awad J.M., Xie G., Qiu W., Muriph R.E. (2020). Sequential decarboxylative [3+2] cycloaddition and Staudinger/aza-Wittig reactions for diastereoselective synthesis of tetrahydro-pyrroloquinazolines and tetrahedro-pyrrolobenzodiazepines. Tetrahedron Lett..

[82] Ma X., Zhang X.F., Awad J.M., Xie G., Qiu W., Zhang W. (2019). One-pot synthesis of tetrahydro-pyrrolobenzodiazepines and tetrahydro-pyrrolobenzodiazepinones through sequential 1,3-dipolar cycloaddition/N-alkylation (N-acylation)/Staudinger/aza-Wittig reactions. Green Chem..

[83] Zhang X.F., Ma X., Qiu W., Evans J., Zhnag W. (2019). Cascade Knoevenagel and aza-Wittig reactions for the synthesis of substituted quinolines and quinolin-4-ols. Green Chem..

[84] Qu F., He P., Hu R., Cheng X., Wang S., Wu J. (2015). Efficient Synthesis of Quinolines via a Knoevenagel/Staudinger/aza-Wittig Sequence. Synth. Commun..

[85] Malkova K., Bubyrev A., Kalinin S., Dar’in D. (2023). Facile access to 3-sulfonylquinolines via Knoevenagel condensation/aza-Wittig reaction cascade involving ortho-azidobenzaldehydes and β-ketosulfonamides and sulfones. Beilstein J. Org. Chem..

[86] Fakhfakh M.A., Fournet A., Prina E., Mouscadet J.F., Franck X., Hocquemiller R., Figadere B. (2003). Synthesis and biological evaluation of substituted quinolines: Potential treatment of protozoal and retroviral co-infections. Bioorg. Med. Chem..

[87] Gopinath V.S., Pinjari J., Dere R.T., Verma A., Vishwakarma P., Shivahare R., Moger M., Kumar Goud P.S., Ramanathan V., Bose P. (2013). Design, synthesis and biological evaluation of 2-substituted quinolines as potential antileishmanial agents. Eur. J. Med. Chem..

[88] Gopaul K., Shintre S.A., Koorbanally N.A. (2015). A Review on the Synthesis and Anti-cancer Activity of 2-substituted Quinolines. Anti-Cancer Agent. Med. Chem..

[89] Swallow S. (2015). Chapter Two—Fluorine in Medicinal Chemistry. Progress Med. Chem..

[90] Ramachandran P.V. (2009). Welcome to ‘Fluorine in Medicinal Chemistry’. Future Med. Chem..

[91] Purser S., Moore P.R., Swallow S., Gouverneur V. (2008). Fluorine in medicinal chemistry. Chem. Soc. Rev..

[92] Gillis E.P., Eastman K.J., Hill M.D., Donnelly D.J., Meanwell N.A. (2015). Applications of Fluorine in Medicinal Chemistry. J. Med. Chem..

[93] Bohm H.J., Banner D., Bendels S., Kansy M., Kuhn B., Muller K., Obst-Sander U., Stahl M. (2004). Fluorine in Medicinal Chemistry. ChemBioChem.

[94] Zhou Y., Wang J., Gu Z., Wang S., Zhu W., Acena J.L., Soloshonok V.A., Izawa K., Liu H. (2016). Next Generation of Fluorine-Containing Pharmaceuticals, Compounds Currently in Phase II–III Clinical Trials of Major Pharmaceutical Companies: New Structural Trends and Therapeutic Areas. Chem. Rev..

[95] Mai A., Rotili D., Tarantino D., Ornaghi P., Tosi F., Vicidomini C., Sbardella G., Nebbioso A., Miceli M., Altucci L. (2006). Small-Molecule Inhibitors of Histone Acetyltransferase Activity:  Identification and Biological Properties. J. Med. Chem..

[96] Goncalves V., Brannigan J.A., Whalley D., Ansell K.H., Saxty B., Holder A.A., Wilkinson A.J., Tate E.W., Leatherbarrow R.J. (2012). Discovery of Plasmodium vivaxN-Myristoyltransferase Inhibitors: Screening, Synthesis, and Structural Characterization of their Binding Mode. J. Med. Chem..

[97] Yu Z., Zheng H., Yuan W., Tang Z., Zhang A., Shi D. (2013). An unexpected one-pot synthesis of multi-substituted quinolines via a cascade reaction of Michael/Staudinger/aza-Wittig/aromatization of ortho-azido-β-nitro-styrenes with various carbonyl compounds. Tetrahedron.

[98] Chaabouni S., Pinkerton N.M., Legrand S., Abid S., Galaup C., Chassaing S., Koivisto J., Hirvonen J., Kostiainen M.A., Bimbo L.M. (2017). Photochemistry of ortho-azidocinnamoyl derivatives: Facile and modular synthesis of 2-acylated indoles and 2-substituted quinolines under solvent control. Synlett.

[99] Kumar G.R., Kumar R., Rajesh M., Reddy M.S. (2018). A nickel-catalyzed anti-carbometallative cyclization of alkyne–azides with organoboronic acids: Synthesis of 2,3-diarylquinolines. Chem. Commun..

[100] Huo Z., Gridnev I.D., Yamamoto Y. (2010). A Method for the Synthesis of Substituted Quinolines via Electrophilic Cyclization of 1-Azido-2-(2-propynyl)benzene. J. Org. Chem..

[101] Wang J., Wang Y.Z., Liu Y., Yan X., Yan Y., Chao S., Shang X., Ni T., Zhou P. (2022). Synthesis of Isoquinolylselenocyanates and Quinolylselenocyanates via Electrophilic Selenocyanogen Cyclization Induced by Pseudohalogen (SeCN)_2_ Generated in situ. Adv. Synth. Catal..

[102] Qiu Y.F., Niu Y.J., Wei X., Cao B.Q., Wang X.C., Quan Z.J. (2019). AgSCF3/Na2S2O8-Promoted Trifluoromethylthiolation/Cyclization of o-Propargyl Arylazides/o-Alkynyl Benzylazides: Synthesis of SCF3-Substituted Quinolines and Isoquinolines. J. Org. Chem..

[103] Chai Z., Zhu Y.M., Yang P.J., Wang S., Wang S., Liu Z., Yang G. (2015). Copper(I)-Catalyzed Kinetic Resolution of N-Sulfonylaziridines with Indoles: Efficient Construction of Pyrroloindolines. J. Am. Chem. Soc..

[104] Kang B., Miller A.W., Goyal S., Nguyen S.T. (2009). Sc(OTf)_3_-catalyzed condensation of 2-alkyl-N-tosylaziridine with aldehydes or ketones: An efficient synthesis of 5-alkyl-1,3-oxazolidines. Chem. Commun..

[105] Ungureanu I., Klotz P., Mann A. (2000). Phenylaziridine as a Masked 1,3 Dipole in Reactions with Nonactivated Alkenes. Angew. Chem..

[106] Wani I.A., Sayyad M., Ghorai M.K. (2017). Domino ring-opening cyclization (DROC) of activated aziridines and epoxides with nitrones via dual-catalysis “on water”. Chem. Commun..

[107] Yi R., Li X., Wan B. (2018). Ring-opening and cyclization of aziridines with aryl azides: Metal-free synthesis of 6-(triflyloxy)quinolines. Org. Chem. Front..

[108] Lin C.H., Tsai M.R., Wang Y.S., Chang N.C. (2003). An Efficient Approach to 3,4-Disubstituted Pyridin-2-ones. Formal Synthesis of Mappicine Ketone. J. Org. Chem..

[109] Wainberg Z.A., Singh A.S., Konecny G.E., McCann K.E., Hecht J.R., Goldman J., Chmielowski B., Finn R.S., O’Brien N., Von Euw E. (2023). Preclinical and Clinical Trial Results Using Talazoparib and Low-Dose Chemotherapy. Clin. Cancer Res..

[110] Wronska N., Majoral J.P., Appelhans D., Bryszewska M., Lisowska K. (2019). Synergistic Effects of Anionic/Cationic Dendrimers and Levofloxacin on Antibacterial Activities. Molecules.

[111] Gentili P.L., Ortica F., Romani A., Favaro G. (2007). Effects of Proximity on the Relaxation Dynamics of Flindersine and 6(5H)-Phenanthridinone. J. Phys. Chem. A.

[112] Papakostas D., Stockfleth E. (2015). Topical treatment of basal cell carcinoma with the immune response modifier imiquimod. Future Oncol..

[113] Xiong J., He H.T., Yang H.Y., Zeng Z.G., Zhong C.R., Shi H., Ouyang M.L., Tao Y.Y., Pang Y.L., Zhang Y.H. (2022). Synthesis of 4-Tetrazolyl-Substituted 3,4-Dihydroquinazoline Derivatives with Anticancer Activity via a One-Pot Sequential Ugi-Azide/Palladium-Catalyzed Azide-Isocyanide Cross-Coupling/Cyclization Reaction. J. Org. Chem..

[114] Kumar R., Arigela R.K., Samala S., Kundu B. (2015). Diversity Oriented Synthesis of Indoloazepinobenzimidazole and Benzimidazotriazolobenzodiazepine from N1-Alkyne-1,2-diamines. Chem. A Eur. J..

[115] Ramachary D.B., Shruthi K.S. (2016). A Brønsted Acid-Amino Acid as a Synergistic Catalyst for Asymmetric List-Lerner-Barbas Aldol Reactions. J. Org. Chem..

[116] Zhang W., Zheng W., Zuo G., Li X., Zhang X.F., Zhang W. (2023). One-pot Mannich/aza-Wittig/deaminative aromatization reactions for the synthesis of 1,2,3,4-tetrahydroacridines and cyclohepta[b]quinolines. New J. Chem..

[117] Sun M., Lu Y., Zhao L., Ding M. (2021). One-pot and divergent synthesis of furo [3,2-c]quinolines and quinazolin-4(3H)-ones via sequential isocyanide-based three-component/Staudinger/aza-Wittig reaction. Tetrahedron.

[118] Wei J., Nie B., Peng R., Cheng X., Wang S. (2016). A Facile Synthesis of Benzofuro [2,3-c]quinolines via a Multicomponent Reaction and Staudinger–Aza-Wittig–Dehydroaromatization Sequence. Synlett.

[119] Bharate J.B., Sharma R., Aravinda S., Gupta V.K., Bharate S.B., Vishwakarma R.A. (2013). Montmorillonite clay catalyzed synthesis of functionalized pyrroles through domino four-component coupling of amines, aldehydes, 1,3-dicarbonyl compounds and nitroalkanes. RSC Adv..

[120] Qu F., Hu R., Gao L., Wu J., Cheng X.H., Wang S., He P. (2015). New and Efficient Synthesis of 2,3,4-Trisubstituted 2H-Pyrrolo [3,4-c]quinolines via a MCR/Staudinger/Aza-Wittig Sequence. Synthesis.

[121] Shi Y., Liao L., He P., Hu Y., Cheng H., Wang S., Wu J. (2016). Synthesis of 2H-pyrrolo [3,4-C]quinoline via an Aldol/Van Leusen/Staudinger/aza-Wittig sequence. Synth. Commun..

[122] Nie B., Wu L., Hu R., Sun Y., Wu J., He P., Huang N. (2017). Synthesis of cyclopropa[c]indeno [1,2-b]quinolines through a MCR/Staudinger/aza-Wittig sequence. Synth. Commun..

[123] Van Es T., Staskun B. (2003). Chlorine- and Sulphur-substituted Pyrrolo [3, 4-b]quinolines and Related Derivatives arising from the Aminolysis of 3, 3, 9-Trichlorothieno [3, 4-b]quinolin-1(3H)-one. S. Afr. J. Chem..

[124] Yi R., Li X., Wan B. (2018). Merging Gold Catalysis and Brønsted Acid Catalysis for the Synthesis of Tetrahydrobenzo[b][1,8]naphthyridines. Adv. Synth. Catal..

[125] Gharpure S.J., Shelke Y.G. (2017). Lewis Acid Mediated Cascade Friedel–Craft/Alkyne Indol-2-yl Cation Cyclization/Vinyl Cation Trapping for the Synthesis of N-Fused Indole Derivatives. Org. Lett..

[126] Costa G.P., Bach M.F., Moraes M.C., Barcellos T., Lenardao E.J., Silva M.S., Alve D. (2020). Sequential Organocatalytic Synthesis of [1,2,3]Triazolo [1,5-a]quinolines. Adv. Synth. Catal..

[127] Alajarin M., Bonillo B., Ortin M.M., Sanchez-Andrada P., Vidal A. (2006). Hydricity-Promoted [1,5]-H Shifts in Acetalic Ketenimines and Carbodiimides. Org. Lett..

[128] Alajarin M., Bonillo B., Ortin M.M., Sanchez-Andrada P., Vidal A. (2011). Tandem 1,5-Hydride Shift/6π Electrocyclization of Ketenimines and Carbodiimides Substituted with Cyclic Acetal and Dithioacetal Functions: Experiments and Computations. Eur. J. Org. Chem..

[129] Ratheesh M., Sindhu G., Helen A. (2013). Anti-inflammatory effect of quinoline alkaloid skimmianine isolated from *Ruta graveolens* L.. Inflamm. Res..

[130] Zuo Y., Pu J., Chen G., Shen W., Wang B. (2019). Study on the activity and mechanism of skimmianine against human non-small cell lung cancer. Nat. Prod. Res..

[131] Wang L.H., Wehland M., Wise P.M., Infanger M., Grimm D., Kreissl M.C. (2023). Cabozantinib, Vandetanib, Pralsetinib and Selpercatinib as Treatment for Progressed Medullary Thyroid Cancer with a Main Focus on Hypertension as Adverse Effect. Int. J. Mol. Sci..

[132] Shen L., Pili R. (2019). Tasquinimod targets suppressive myeloid cells in the tumor microenvironment. Oncoimmunology.

[133] Hong D.S., Rosen P., Lockhart A.C., Fu S., Janku F., Kurzrock R., Khan R., Amore B., Caudillo I., Deng H. (2015). A first-in-human study of AMG 208, an oral MET inhibitor, in adult patients with advanced solid tumors. Oncotarget.

[134] Yuan S., Peng L., Liu Y., Till B.G., Yan X., Zhang J., Zhu L., Wang H., Zhang S., Li H. (2023). Low-dose anlotinib confers improved survival in combination with immune checkpoint inhibitor in advanced non-small cell lung cancer patients. Cancer Immunol. Immunother..

[135] Zhao R., Zou W., Zhao X. (2023). Treatment of neurofibromatosis type II with anlotinib: A case report and literature review. Anti-Cancer Drugs.

[136] He R., Lin F., Yu B., Qiu J., Zheng L. (2022). The efficacy and adverse events of delafloxacin in the treatment of acute bacterial infections: A systematic review and meta-analysis of randomized controlled trials. Front. Pharmacol..

[137] Gould R.G., Jacobs W.A. (1939). The Synthesis of Certain Substituted Quinolines and 5,6-Benzoquinolines. J. Am. Chem. Soc..

[138] Jentsch N.G., Hume J.D., Crull E.B., Beauti S.M., Pham A.H., Pigza J.A., Kessl J.J., Donahue M.G. (2018). Quinolines from the cyclocondensation of isatoic anhydride with ethyl acetoacetate: Preparation of ethyl 4-hydroxy-2-methylquinoline-3-carboxylate and derivatives. Beilstein J. Org. Chem..

[139] Gharpure S.J., Nanda S.K., Adate P.A., Shelke Y.G. (2017). Lewis Acid Promoted Oxonium Ion Driven Carboamination of Alkynes for the Synthesis of 4-Alkoxy Quinolines. J. Org. Chem..

[140] Hande P.E., Mishra M., Ali F., Kapoor S., Datta A., Gharpure S.J. (2020). Design and Expeditious Synthesis of Quinoline-Pyrene-Based Ratiometric Fluorescent Probes for Targeting Lysosomal pH. ChemBioChem.

[141] Gharpure S.J., Nanda S.K., Fartade D.J. (2021). Formal [4+2] Cycloaddition of o-Aza-Quinone Methide for the Synthesis of 1,4-Heterocycle-Fused Quinolines. Adv. Synth. Catal..

[142] Su H., Bao M., Pei C., Hu W.H., Qiu L.H., Xu X.F. (2019). Gold-catalyzed dual annulation of azide-tethered alkynes with nitriles: Expeditious synthesis of oxazolo [4,5-c]quinolines. Org. Chem. Front..

[143] Su H., Bao M., Huang J.J., Qiu L.H., Xu X.F. (2019). Silver-Catalyzed Carbocyclization of Azide-Tethered Alkynes: Expeditious Synthesis of Polysubstituted Quinolines. Adv. Synth. Catal..

[144] Huang J., Su H., Bao M., Qiu L., Zhang Y., Xu X. (2020). Gold(iii)-catalyzed azide-yne cyclization/O–H insertion cascade reaction for the expeditious construction of 3-alkoxy-4-quinolinone frameworks. Org. Biomol. Chem..

[145] Lenoci A., Tomassi S., Conte M., Benedetti R., Rodriguez V., Carradori S., Secci D., Castellano S., Sbardella G., Filetici P. (2014). Quinoline-Based p300 Histone Acetyltransferase Inhibitors with Pro-apoptotic Activity in Human Leukemia U937 Cells. ChemMedChem.

[146] Zhao S., Zhou W., Liu J. (2009). Synthesis and HMG-CoA reductase inhibition of 2-cyclopropyl-4-thiophenyl-quinoline mevalonolactones. Bioorg. Med. Chem..

[147] Ma X., Qiu W., Liu L., Zhang X., Awad J.M., Evans J., Zhang W. (2021). Synthesis of tetrahydropyrrolothiazoles through one-pot and four-component N,S-acetalation and decarboxylative [3+2] cycloaddition. Green Synth. Catal..

[148] Ward S.A. (1988). Mechanisms of chloroquine resistance in malarial chemotherapy. Trends Pharmacol. Sci..

[149] Homewood C.A., Warhurst D.C., Peters W., Baggaley V.C. (1972). Lysosomes, pH and the Anti-malarial Action of Chloroquine. Nature.

[150] Kim T.H., Kim H.K., Hwang E.S. (2017). Novel anti-adipogenic activity of anti-malarial amodiaquine through suppression of PPARγ activity. Arch. Pharm. Res..

[151] Brummendorf T.H., Cortes J.E., Milojkovic D., Gambacorti-Passerini C., Clark R.E., le Coutre P., Garcia-Gutierrez V., Chuah C., Kota V., Lipton J.H. (2022). Bosutinib versus imatinib for newly diagnosed chronic phase chronic myeloid leukemia: Final results from the BFORE trial. Leukemia.

[152] Mezger K., Ebert S., Muhle H.E., Stadt U.Z., Borkhardt A., Dilloo D., Faber J., Feuchtinger T., Imschweiler T., Jorch N. (2022). Amsacrine combined with etoposide and methylprednisolone is a feasible and safe component in first-line intensified treatment of pediatric patients with high-risk acute lymphoblastic leukemia in CoALL08-09 trial. Pediatr. Blood Cancer.

[153] Rizzo A., Dalia Ricci A., Brandi G. (2022). Neoadjuvant Dovitinib in Early- and Intermediate-Stage Hepatocellular Carcinoma. Oncologyst.

[154] Thomas K.D., Adhikari A.V., Shetty N.S. (2010). Design, synthesis and antimicrobial activities of some new quinoline derivatives carrying 1,2,3-triazole moiety. Eur. J. Med. Chem..

[155] Lin A.J., Loo T.L. (1978). Synthesis and antitumor activity of halogen-substituted 4-(3,3-dimethyl-1-triazeno)quinolines. J. Med. Chem..

[156] Lavrard H., Larini P., Popowycz F. (2017). Superacidic Cyclization of Activated Anthranilonitriles into 2-Unsubstituted-4-aminoquinolines. Org. Lett..

[157] Vidyacharan S., Sagar A., Sharada D.S. (2015). A new route for the synthesis of highly substituted 4-aminoquinoline drug like molecules via aza hetero–Diels–Alder reaction. Org. Biomol. Chem..

[158] Zhang X.F., Ma X.M., Qiu W., Award J.M., Evans J., Zhang W. (2020). One-Pot Mannich, Aza-Wittig and Dehydrofluorinative Aromatization Reactions for Direct Synthesis of 2,3-Disubstituted 4-Aminoquinolines. Adv. Synth. Catal..

